# Online Scheduling and the Digital Front Door to Unequal Care

**DOI:** 10.2196/108179

**Published:** 2026-08-31

**Authors:** Joseph H Joo, Chieh-Liang Wu, Joshua M Liao

**Affiliations:** 1 Department of Internal Medicine, The University of Texas Southwestern Medical Center, 5323 Harry Hines Blvd, Dallas, TX, 75390, United States, 1 2146483111; 2 Taichung Veterans General Hospital, Taichung, Taiwan, Taiwan; 3 Ascension, St. Louis, MO, United States

**Keywords:** digital health, health technology, care engagement

## Abstract

Online appointment platforms are often viewed as tools of convenience, but they may reproduce and potentially amplify inequities rooted in underlying insurance and reimbursement structures. In this commentary, we highlight implications from a study using simulated profiles of patients with statutory health insurance (SHI) and private health insurance (PHI) to conduct an internet-based audit of online appointments. Not only was the time to the first available appointment longer for SHI than PHI patients, but also the PHI profile had access to more listings with appointments, and listings offering earlier PHI than SHI appointments appeared higher in search results than listings offering the same appointment timing to both. Accounting for strengths, limitations, and opportunities for future research, the study suggests several policy implications. First, leaders should view online scheduling platforms as access infrastructure, not merely convenience tools, and evaluate them with respect to access and disparities, not only adoption and satisfaction. Second, future evaluation and work should treat access as multidimensional, assessing multiple measures of access and comparing online with other scheduling channels. Third, insurance and delivery reform should accompany actions to govern digital platforms, which can organize and display, but cannot alone eliminate, disparities rooted in reimbursement and care structures. Ultimately, the relevant question about online scheduling platforms is not only whether such systems make booking easier but also whether they narrow, preserve, or widen broader health system inequalities. Digital front doors should be judged both by how easily they open and whether patients have equitable opportunities to pass through them.

Online appointment platforms are often viewed as tools of convenience. However, while these systems can reduce friction in finding care, convenience does not necessarily mean equitable access. Problematically, online platforms may reproduce and potentially amplify health system inequities rooted in underlying structures such as insurance and reimbursement systems.

The study by Kreuzenbeck and Angerer [1] illustrates this risk. The authors conducted a cross-sectional, internet-based audit of appointments displayed on an online platform in Berlin using simulated profiles representing patients with statutory health insurance (SHI) and private health insurance (PHI). Of 1867 listings screened, 492 offered at least one bookable appointment to both profiles and were included in the paired waiting time analysis. On average, the time to the first available appointment was approximately 4 weeks longer for patients with SHI than PHI; after excluding the longest waits, the difference remained over 3 weeks. The PHI profile also had access to more than twice as many listings with appointments as the SHI profile. In an exploratory ranking analysis of 4 specialties, listings offering earlier PHI than SHI appointments appeared higher in PHI search results than listings offering the same appointment timing to both profiles. Among 175 listings, those offering equal SHI and PHI timing appeared an average of 58 positions—nearly 3 result pages—lower.

## Strengths

The study has strengths. It evaluated access via online scheduling interfaces where patients increasingly encounter the health system. Its within-listing comparison reduced confounding from stable differences between practices, while switching the insurance status assigned to profiles for certain listings in each specialty supported an interpretation that availability followed insurance status rather than age or gender. The ranking analysis also raised an important question about whether platform visibility could reinforce differential appointment availability.

At a fundamental level, difficulty obtaining timely appointments reflects limited or unevenly allocated clinical capacity relative to demand. Digital platforms can reduce the burden of searching for available capacity, but they cannot create clinician supply or remove the financial and organizational incentives affecting how providers allocate appointments. In turn, the study’s main contribution is not underscoring that patients with PHI receive earlier appointments—such differences have been observed through conventional booking and are plausibly related to differential reimbursement [2]. Instead, the study by Kreuzenbeck and Angerer [1] illustrates how disparities can manifest digitally, and how platform filters and rankings may organize, surface, and potentially amplify disparities originating elsewhere in the health care system.

## Limitations and Areas for Future Research

Several limitations should temper interpretation. First, the study examined one platform using a proprietary algorithm in one city while omitting telephone, referral, or in-person access, collectively limiting the representativeness of study results. Second, the principal outcome was days until the first displayed appointment—a useful but incomplete measure that does not capture full, realized access. For example, 2 listings could each show a PHI appointment 10 days away and an SHI appointment 40 days away. Yet 1 listing might offer only 1 appointment to each group, whereas the other might offer numerous additional PHI appointments during the intervening period. These scenarios would yield the same difference in time to first appointment while representing very different access environments. Total number of available slots, distribution over time, or appointment flexibility can more fully capture those environments but were not measured in the Kreuzenbeck and Angerer [1] study.

Third, paired analyses were restricted to listings offering appointments to both SHI and PHI profiles. This supported clean within-provider comparisons but primarily addressed the intensive margin: waiting time differences when both profiles had a bookable appointment. Although the study described a substantial extensive margin gap, with the number of listings with offerings differing markedly by insurance status, reported estimates should not be interpreted as a complete measure of access gaps.

These limitations signal valuable opportunities for future research. Studies could compare online and conventional booking within SHI and PHI separately while accounting for differences in age, digital literacy, clinical urgency, and flexibility among patients selecting each channel. Longitudinal analyses could compare the SHI-PHI gap before and after practices adopt online scheduling, ideally using comparable nonadopting practices as controls. Such designs could offer insight about if and how online booking affects pre-existing disparities.

## Policy Implications

Nonetheless, the study suggests several policy implications. First, leaders should view online scheduling platforms as access infrastructure, not merely convenience tools. Online systems can reduce waiting times and administrative burden, but benefits do not necessarily accrue equally [3,4]. As digital platforms become more central to patient decisions, platform filters, visibility, and rankings become part of access architecture [1,4,5]. Platforms should be evaluated with respect to access and disparities, not only adoption and satisfaction.

Second, future evaluation and work should treat access as multidimensional [6]. Leaders need information about not only time to first appointment but also bookable providers, waiting time distributions, appointment slot density, geographic accessibility, booking flexibility, and stability of availability over time [1,5,6]. Future evaluations should also directly assess both intensive and extensive margins and compare online with other scheduling channels.

Third, insurance and delivery reform should accompany actions to govern digital platforms, which can organize and display, but cannot alone eliminate, disparities rooted in reimbursement and care structures [1,2,4]. For instance, comparing Germany’s dual system to others (eg, Taiwan’s single-payer system or the US multi-payer system) may help distinguish inequalities associated with payer status from barriers that persist because of provider capacity, institutional demand, patient preferences, or other factors [7,8]. Digital platform effects must be interpreted and addressed within the financing and delivery systems in which they operate.

## Conclusion

Increasingly, online scheduling platforms mediate how patients encounter scarce health care capacity. For leaders, the relevant question is not only whether such systems make booking easier but also whether they narrow, preserve, or widen inequalities rooted in the broader health system. Digital front doors should be judged both by how easily they open and whether patients have equitable opportunities to pass through them.

## Abbreviations

PHI: private health insurance
SHI: statutory health insurance
